# THE ROLE OF NEIGHBORHOOD DISADVANTAGE IN PAIN AND MEDICATION AMONG OLDER ADULTS WITH OR WITHOUT COGNITIVE IMPAIRMENT

**DOI:** 10.1093/geroni/igae098.0596

**Published:** 2024-12-31

**Authors:** Yulin Yang, Ashwin Kotwal, Lauren Hunt, Scarlett Gomez, Jacqueline Torres

**Affiliations:** University of California San Francisco, San Francisco, California, United States; University of California San Francisco Medical Center, San Francisco, California, United States; University of California San Francisco, San Francisco, California, United States; University of California San Francisco, San Francisco, California, United States; University of California San Francisco, San Francisco, California, United States

## Abstract

Neighborhoods are vital to older adults’ health and well-being, yet how they influence pain outcomes and medication use among older adults, with or without cognitive impairment, remains unknown. We evaluated the neighborhood effects on pain outcomes and medication use by cognitive impairment status. We use two distinct neighborhood socioeconomic status measures – disadvantage (including poverty, unemployment, female-headed families, households receiving public assistance income, and proportion of African Americans) and affluence (including adults with a college education, incomes>75K, and employed in managerial and professional occupations). We analyze data from 9,351 older respondents in the Health and Retirement Study from 4,611 census tracts. We fit multilevel logistic regression models with random intercepts to evaluate the neighborhood effects on the risk of reporting pain, pain-related disability, over-the-counter (OTC) pain medication, and opioid medication. In the overall sample, neighborhood affluence is associated with lower odds of pain and pain-related disability, and higher odds of using OTC medication. Among 6,675 cognitively normal respondents, neighborhood disadvantage is associated with greater pain-related disability, while neighborhood affluence is associated with lower odds of pain, pain-related disability, and higher odds of using OTC medication. Among 2,676 respondents with cognitive impairment, neither neighborhood disadvantage nor affluence is associated with any pain outcomes and medication. Neighborhood affluence predicted higher odds of pain-related disability; however, the 95% confidence interval crossed the null. These findings suggest that neighborhood factors are important social determinants of pain outcomes and management among older adults with and without cognitive impairment.

